# Reimagining Arterial Hypertension and Dyslipidemia Care: Telemedicine’s Promise and Pitfalls from the Slovak Patient Viewpoint

**DOI:** 10.3390/clinpract15110197

**Published:** 2025-10-27

**Authors:** Stefan Toth, Adriana Jarolimkova, Patrik Bucek, Martin Sevcik, Pavol Fulop, Tibor Poruban

**Affiliations:** 1 Cardiology Outpatient Clinic, Kardiocomp S.r.o., Letna 45, 040 01 Kosice, Slovakia; 2 SLOVACRIN & MEDIPARK, Faculty of Medicine, Pavol Jozef Safarik University, Trieda SNP 1, 040 11 Kosice, Slovakia; 3 Department of Infectious Diseases, University Hospital of Jan Adam Rayman, 080 01 Presov, Slovakia; 4 East Slovak Institute of Cardiovascular Diseases and School of Medicine, Pavol Jozef Safarik University, 040 01 Kosice, Slovakia; fulop.pavol@gmail.com

**Keywords:** barriers and opportunities, arterial hypertension, dyslipidemia, questionnaire study, Slovakia, telemonitoring

## Abstract

**Background and objectives**: Numerous studies and meta-analyses have established the efficacy of telemonitoring for blood pressure and other components of metabolic syndrome in improving disease management. Nevertheless, the adoption of telemonitoring technologies is often hindered by personal, technological, and systemic barriers. In Slovakia, where patient–physician contact rates are high, there is limited research on patients’ perspectives regarding telemedicine adoption for cardiovascular risk management. The objective of this study was to examine patients’ perspectives on and perceived obstacles to the use of telemonitoring for arterial hypertension and dyslipidemia in Slovakia. **Methods**: This cross-sectional, questionnaire-based survey targeted a cohort of 18,053 patients. The survey instrument was designed to gather data on several key areas: patient demographic characteristics, blood pressure measurement habits, the utilization of smart technologies, perceived benefits and barriers to telemonitoring, and patients’ knowledge of their lipid profiles and cardiovascular risk factors. Statistical analysis included chi-square tests, ANOVA, and effect size calculations with 95% confidence intervals (CI). **Results**: A total of 1787 patient responses (9.9%) were collected. Among the respondents, 67.4% (*n* = 1204) had arterial hypertension, while 7.9% (*n* = 95) were on non-pharmacological therapy. Only 21.2% (*n* = 255) of hypertensive patients measured their blood pressure daily, with a significantly higher proportion of men than women (28.6% vs. 12.7%, *p* = 0.011, Cohen’s d = 0.42). The most frequent users of blood pressure monitoring were in the 31–45 age group (*p* = 0.001, η^2^ = 0.08). A total of 19.4% (*n* = 347) of respondents used wearable devices, and 6.3% (*n* = 113) used blood pressure monitors connected to an application. Smart technology use was significantly more common in the 31–45 age group (*p* = 0.01, Cramer’s V = 0.15). Moderate interest in telemedicine was expressed by 69.8% (*n* = 1247) of respondents, though only 27.4% (*n* = 490) showed strong interest. The majority of patients (73.8%, *n* = 1319) did not know their LDL-C levels, and 45.7% (*n* = 817) of those who did had elevated levels. **Conclusions**: The findings suggest that while interest in telemedicine methods for the management of arterial hypertension and dyslipidemia exists among Slovak patients, it is more moderate than initially assumed. Importantly, expressed willingness to participate in a study should not be directly equated with readiness to adopt new technologies in daily practice. Successful integration of telemonitoring into the Slovak healthcare system will therefore require not only patient engagement but also active support from healthcare providers to overcome practical and motivational barriers. These findings highlight the need for targeted implementation strategies that address the specific barriers identified in the Central and Eastern European healthcare context.

## 1. Introduction

Arterial hypertension is a widespread global health issue, affecting approximately 30–40% of the adult population worldwide. Despite the availability of effective pharmacotherapies and frequent medical care, especially in developed countries, blood pressure control rates remain suboptimal [1,2]. Recent data indicates that only about 23% of women and 18% of men with hypertension successfully achieve blood pressure levels below 140/90 mmHg. In certain regions, particularly Central and Eastern Europe, these control rates are even lower. The most comprehensive pooled analysis to date, including over 100 million participants, found that the age-standardized prevalence of hypertension among adults aged 30–79 years was 34% for women and 32% for men in 2019, with rates exceeding 50% in some central and eastern European countries [1].

European Society of Cardiology (ESC) and European Hypertension Society (EHS) recommend a target blood pressure of less than 130/80 mmHg for most adults. However, treatment goals should be tailored to individual patients based on factors such as age and the presence of co-existing conditions. Even a modest reduction of just 10 mmHg in systolic blood pressure can significantly lower the risk of major cardiovascular events, including stroke and cardiovascular mortality, by 20–30% [2]. Even modest reductions in blood pressure confer significant risk reduction, as supported by large-scale meta-analyses [2,3].

Home blood pressure monitoring (HBPM) is strongly recommended by current hypertension guidelines, including those from the ESC, because it provides more accurate and objective readings than office measurements and has superior prognostic value for cardiovascular outcomes, including stroke and mortality. HBPM is essential for identifying white-coat and masked hypertension, monitoring therapy, and guiding clinical decision-making [4,5,6].

Both hypertension and dyslipidemia are the most common modifiable risk factors for atherosclerotic cardiovascular disease. It is important to note that the term “lipitensia” is not a recognized medical term; the correct scientific term is the coexistence of hypertension and dyslipidemia [3,7].

Telemedicine and other digital health interventions, particularly blood pressure telemonitoring, have shown promising results. Multiple meta-analyses and clinical trials have demonstrated that these technologies can improve blood pressure control, lead to greater reductions in both systolic and diastolic pressure, and enhance patient adherence to treatment plans [8,9,10]. Telemonitoring consistently leads to greater reductions in systolic and diastolic compared to usual care with pooled data showing telemonitoring interventions reduce systolic BP by 3–7 mmHg and diastolic BP by 2–5 mmHg, and increase the proportion of patients achieving BP control by 10–15% over standard care [11,12,13,14,15,16].

However, widespread adoption still faces several barriers, including organizational barriers such as lack of standardized protocols and limited integration with electronic health records, economic barriers including high initial costs and reimbursement uncertainties, and technical barriers such as variable internet connectivity and digital literacy gaps [17,18,19,20,21,22,23,24].

Despite high rates of patient blood pressure and higher rates of target blood pressure achievement cent-physician interactions in countries like Slovakia, Japan, and South Korea, traditional outpatient models have often fallen short of achieving satisfactory hypertension control [1,25]. While numerous meta-analyses have demonstrated the effectiveness of telemedicine, most have focused on large healthcare systems in Western Europe and the United States. There is limited research that takes into account the unique healthcare systems and patient characteristics of Central and Eastern Europe. In particular, in Slovakia, where the frequency of face-to-face consultations between patients and doctors is relatively high, there are no systematic studies investigating patients’ expectations and perceived barriers to telemedicine.

Previous research has primarily focused on the technical and clinical components of Learning Health Systems (LHS), such as data infrastructure, patient outcomes, and implementation processes. However, relatively little attention has been paid to the organizational culture that underpins the successful functioning of LHS. Organizational culture shapes how knowledge is shared, how stakeholders collaborate, and how innovations are adopted or resisted. Without a culture that supports learning, even the most advanced infrastructures risk failure. This gap in the literature highlights the need to examine the cultural dimension as a determinant of LHS success, particularly in healthcare contexts where systemic change is often hindered by entrenched practices.

Given these factors, the primary aim of this study was to evaluate the overall patient acceptance of telemedicine for managing the coexistence of hypertension and dyslipidemia in Slovakia. This study aims to fill this research gap in the Central and Eastern European context and provide concrete insights that will contribute to future healthcare strategies. The research sought to identify key obstacles and opportunities for its broader adoption into routine clinical practice, providing valuable insights for future healthcare strategies. The study also focused on assessing patients’ general habits in managing hypertension and dyslipidemia.

## 2. Materials and Methods

### 2.1. Study Population

This study was conducted as a cross-sectional, questionnaire-based survey between October and December 2024. The survey population was drawn from the patient registry of the cardiology outpatient clinic *Kardiocomp s.r.o.*, located in Eastern Slovakia. The clinic’s total patient base comprises 18,053 individuals, with a majority from Eastern Slovakia and a smaller subset of approximately 2500 patients from Central Slovakia and 1000 from Western Slovakia.

The questionnaire was administered to the target population via email, and responses were securely collected using an online survey platform. Participants were recruited through email invitations sent to addresses on the clinic’s patient database. To maximize the response rate, two reminder emails were distributed at two-week intervals. Participation was strictly voluntary. Inclusion criteria were 1. confirmed medical diagnosis of arterial hypertension and/or dyslipidemia; 2. at least 18 years of age; 3. residency in Slovakia. Exclusion criteria included inability to provide informed consent, cognitive or physical impairments that would prevent questionnaire completion, and patients without valid email addresses. An informed consent was obtained from all participants. A principal investigator of the study was S.T.

Power analysis indicated that a minimum sample size of 377 participants would be required to detect medium effect sizes (Cohen’s d = 0.5) with 80% power at α = 0.05. Despite the low response rate (9.9%), the achieved sample size of 1787 participants provided adequate power for the planned analyses. The low response rate was anticipated given the digital nature of survey distribution and was addressed through multiple reminder strategies and comprehensive bias discussion in limitations.

### 2.2. Questionnaire Development and Data Processing

The structured questionnaire was meticulously developed through a comprehensive review of existing literature and expert consultations with specialists in cardiology, telemedicine, and health informatics. To ensure clarity, relevance, and face validity, a pilot study was conducted with a sample of 10 patients. Feedback from this pilot was used to refine and optimize the final questionnaire. Internal consistency reliability was assessed using Cronbach’s alpha, which ranged from 0.72 to 0.84 across different questionnaire sections, indicating acceptable to good reliability. To ensure rigor and transparency in classifying studies as LHS, the inclusion criteria were derived through an iterative process: first, a targeted review of conceptual and empirical papers on LHS to identify recurring defining characteristics; second, refinement through consultation with two external experts in health systems research to enhance content validity; and third, piloting on a sample of 20 articles with consensus resolution of disagreements. This process strengthens reliability and reduces the risk of selective inclusion. Additionally, while the primary screening will be conducted by SY, independent double screening of a random 10% sample by GB will be performed at both title/abstract and full-text stages. Inter-rater agreement will be calculated (Cohen’s kappa), and disagreements resolved through discussion or a third reviewer. This structured approach increases transparency and mitigates potential bias in study selection and coding.

The final survey instrument comprised six distinct sections:

**1. Demographic and Background Information:** This section collected data on participant age (categorized as 18–30, 31–45, 46–65, 66–75, >76 years), gender (male/female), geographical region of residence (Eastern/Central/Western Slovakia), and educational level (primary, secondary, university).

**2. Hypertension Diagnosis and Measurement Practices:** This section assessed participants’ confirmed diagnosis of hypertension (yes/no), current treatment status (pharmacological/non-pharmacological/none), and blood pressure measurement frequency (daily, several times per week, weekly, less than weekly, never). Questions included: “How often do you measure your blood pressure at home?” and “What type of device do you use for blood pressure measurement?”.

**3. Acceptance of Telemedicine:** This section gauged participants’ willingness to adopt telemedicine using questions such as: “Would you be interested in participating in physician-supervised blood pressure telemonitoring?” (very interested/somewhat interested/neutral/not interested/strongly opposed) and “What features would be most important to you in a telemedicine system?” (multiple choice options).

**4. Perceived Benefits of Smart Technologies:** This section utilized a 5-point Likert scale (1 = strongly disagree to 5 = strongly agree) to assess perceived usefulness. Example items included: “Sharing blood pressure data with my physician would be very useful” and “Smart technology would make blood pressure monitoring easier.” An “unable to determine” option was provided for all items.

**5. Barriers and Facilitators to Adoption:** This section identified factors that would encourage or hinder telemedicine implementation through questions such as: “What barriers prevent you from using smart technologies for health monitoring?” (cost, complexity, privacy concerns, preference for traditional methods, other) and “What would motivate you to use telemedicine services?” (open-ended responses).

**6. Knowledge of Cardiovascular Risk:** This section evaluated participants’ knowledge through specific questions: “Do you know your current LDL cholesterol level?” (yes/no, with numerical value if yes), “Are you interested in remote cardiovascular risk assessment?” (yes/no), and “How would you rate your overall cardiovascular risk?” (low/moderate/high/don’t know).

Data cleaning procedures included identification and removal of incomplete responses (less than 80% completion), detection of outliers using interquartile range methods, and verification of logical consistency across responses. Categorical variables were coded numerically for analysis, and continuous variables were checked for normality using Shapiro–Wilk tests.

### 2.3. Statistical Analysis

Sample characteristics were analyzed using descriptive statistics. Continuous variables are presented as means with standard deviations, while categorical variables are presented as frequencies and percentages with sample sizes (*n*). Changes in the monitored parameters were evaluated using analysis of variance for repeated measures (ANOVA) and Tukey’s post hoc test. Pearson’s correlation coefficient was used to determine correlations between parameter changes in the early period and at later time intervals. All analyses were performed using two-sided tests at a 5% significance level (*p*-values < 0.05 were considered statistically significant). Comparisons of continuous parameter means across two or more groups were performed using analysis of variance (ANOVA). The chi-square (χ^2^) test was used to compare categorical variables. A paired t-test was used to determine the effect of treatment by comparing parameter values. Effect sizes were calculated using Cohen’s d for *t*-tests, eta-squared (η^2^) for ANOVA, and Cramer’s V for chi-square tests. Multivariate logistic regression analyses were performed to identify independent predictors of telemedicine acceptance, controlling for age, gender, education level, and hypertension status. The data collected in this study were processed and analyzed using SPSS version 29.0 for Windows (IBM Corp, 2022. IBM SPSS, 29.0 Armonk, NY, USA). Quantitative data were analyzed using descriptive statistics and statistical hypothesis testing methods. Each parameter was expressed as the mean (MEAN) with a standard deviation (SD). Absolute and relative frequencies were used for categorical variables.

## 3. Results

A total of 18,053 patients with valid email addresses were contacted, resulting in 1787 responses, which represents a 9.9% response rate. The cohort was almost evenly distributed by sex, with 53.17% (*n* = 950) female respondents and 46.83% (*n* = 837) male respondents.

Regarding age distribution, the most represented age group was 46–65 years (48.81%; *n* = 872), followed by 31–45 years (23.02%; *n* = 411), and 65–75 years (15.87%; *n* = 287). Less represented groups included young adults aged 18–30 years (5.56%; *n* = 99) and the elderly over 76 years (6.75%; *n* = 118).

**Figure 1:**
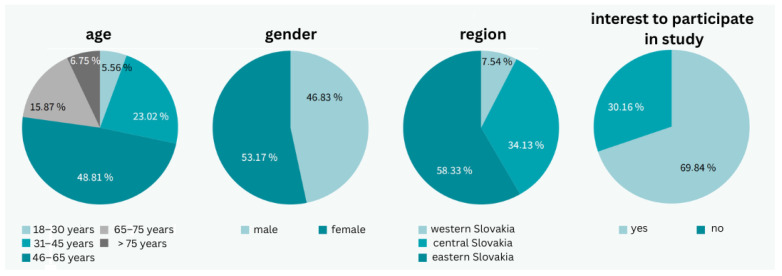
General characteristics of the patient group (photo: authors).

Geographically, the majority of respondents were from Eastern Slovakia (58.33%; *n* = 1042), followed by Central Slovakia (34.13%; *n* = 610), and Western Slovakia (7.54%; *n* = 135). Overall, 69.8% (*n* = 1247) of respondents expressed an interest in participating in physician-supervised telemonitoring, though only 27.4% (*n* = 490) showed strong interest (Figure 1).

Among all respondents, 67.4% (*n* = 1204) had a confirmed diagnosis of hypertension, with 7.9% (*n* = 95) managing their condition with non-pharmacological therapy. An additional 24.3% (*n* = 434) had dyslipidemia only, and 8.3% (*n* = 149) had both conditions confirmed. Only 17.5% (*n* = 211) of all respondents measured their blood pressure daily, while 40.1% (*n* = 483) measured it less than once a week, and 4.4% (*n* = 53) never measured their blood pressure.

Among hypertensive patients, only 21.2% (*n* = 255) measured their blood pressure daily, 28.24% (*n* = 340) several times a week, 21.2% (*n* = 255) once a week, 28.82% (*n* = 347) less frequently, and 0.6% (*n* = 7) never measured their blood pressure. Men were significantly more likely to measure blood pressure daily (25.42%; *n* = 306) compared to women (11.29%; *n* = 136) (*p* = 0.004). Among hypertensive patients, 28.6% (*n* = 344) of men measured blood pressure daily compared to 12.7% (*n* = 153) of women (*p* = 0.011, Cohen’s d = 0.42). Older age was strongly associated with more frequent daily monitoring (*p* = 0.001, η^2^ = 0.08). Diagnosis and prescription of antihypertensive therapy were significantly associated with daily blood pressure measurement (*p* = 0.003, Cramer’s V = 0.18), while diagnosis without therapy was not (*p* = 0.127), and lack of diagnosis or unawareness correlated negatively (*p* = 0.025, Cramer’s V = 0.12). Multivariate regression analysis identified significant independent predictors of daily blood pressure monitoring: male gender (OR = 2.1, 95% CI: 1.6–2.8, *p* < 0.001), age > 65 years (OR = 1.8, 95% CI: 1.3–2.5, *p* < 0.001), and pharmacological treatment (OR = 1.6, 95% CI: 1.2–2.1, *p* = 0.002).

**Figure 2:**
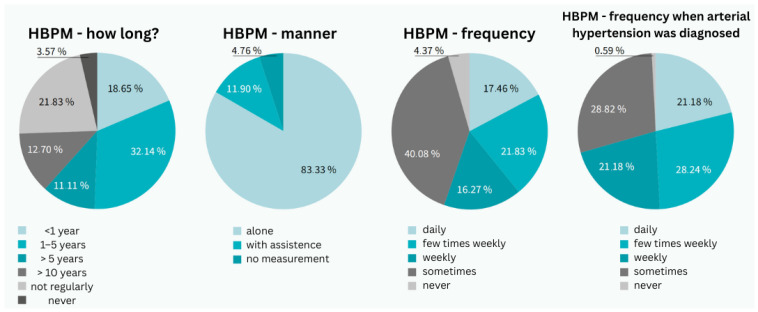
Characteristics of the patient group from a perspective of arterial hypertension (photo: authors). HBPM—home blood pressure measurement.

Most patients (83.3%; *n* = 1488) measured blood pressure independently, while 11.9% (*n* = 213) required assistance. The majority had been monitoring their blood pressure for 1–5 years (32.1%; *n* = 574), while only 18.7% (*n* = 334) had been measuring it for less than a year (Figure 2).

A total of 71.4% (*n* = 1276) used a digital cuff at home, 20.6% (*n* = 368) used a manual sphygmomanometer, 6.3% (*n* = 113) used a monitor connected to a mobile application, and 19.4% (*n* = 347) used wearable smart devices. Overall, 28.6% (*n* = 511) used smart applications, smartwatches, or other devices.

**Figure 3:**
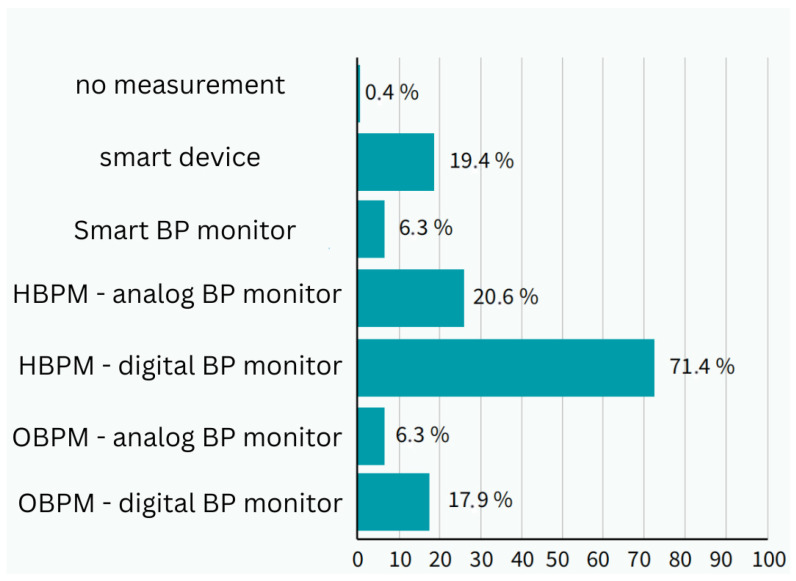
Method of measuring blood pressure in respondents and use of smart technologies in measurement (photo: authors). BP—blood pressure; HBPM—home blood presure measurement; OBPM—office blood pressure measurement.

Smart technology was most commonly used by patients aged 31–45 years, where its use was significantly higher than other methods (*p* = 0.01, Cramer’s V = 0.15). Among users of smart technology, 83.3% (*n* = 426) used smartwatches, 20.8% (*n* = 106) used mobile applications alone or in combination, and 12.5% (*n* = 64) used an intelligent blood pressure monitor. Daily use was reported by 50% (*n* = 256), whereas 27.8% (*n* = 142) used it less than once a week or occasionally (Figure 3).

Among respondents, 43.65% (*n* = 780) found data sharing with a physician to be very useful, 36.50% (*n* = 652) valued continuous and simplified monitoring, and 43.65% (*n* = 780) found the ability to store blood pressure values beneficial. Only 2.77% (*n* = 49) considered notifications for blood pressure measurement and medication reminders to be unhelpful. Regarding barriers, 66.3% (*n* = 1185) reported no obstacles to smart technology use, 16.7% (*n* = 298) mentioned cost, and 19.8% (*n* = 354) preferred traditional methods.

A strong interest in smart technology for blood pressure measurement was expressed by 27.4% (*n* = 490), primarily in the 18–30 age group, followed by 31–45-year-olds. Additionally, 40.5% (*n* = 724) were somewhat interested. Lack of interest or reluctance was expressed by 7.9% (*n* = 141), with the highest prevalence in patients over 75 years (23.5%; *n* = 420), followed by the 65–75 age group (10%; *n* = 179), 31–45 years (6.9%; *n* = 123), 45–65 years (5.7%; *n* = 102), and the lowest in the 18–30 age group (7.1%; *n* = 127).

Age-related disparities in technology acceptance were particularly pronounced. Patients over 75 years showed significantly lower interest in telemedicine (23.5% expressing reluctance vs. 7.1% in 18–30 age group, *p* < 0.001). This disparity may be attributed to lower digital literacy, increased comfort with traditional healthcare models, and potential concerns about technology complexity in older adults.

Most patients found setting up and connecting smart devices to applications to be difficult, whereas linking to applications and device maintenance (charging) was considered easy. The complexity of setup was significantly correlated with age (*p* < 0.001), with the 18–45 age group finding it easiest. Similarly, connecting to applications was easiest for the 31–45 age group, followed by 18–30-year-olds. The most requested features included a user-friendly interface (68.7%; *n* = 1228), personalized reminders, and physician feedback without requiring a clinic visit (52.4%; *n* = 936).

Overall, 73.8% (*n* = 1319) were unaware of their LDL-C levels. Among those who knew their LDL-C values, 45.7% (*n* = 817) had elevated levels (assuming a hypothetical classification of low cardiovascular risk), and four patients had LDL-C levels ranging between 5 and 8.2 mmol/L. The average LDL-C value was 3.77 ± 1.75 mmol/L.

A total of 88.5% (*n* = 1581) expressed interest in calculating their cardiovascular risk, with interest strongly associated with hypertension diagnosis and therapy (*p* = 0.025, Cramer’s V = 0.14) and being more prevalent in women than men (*p* = 0.003, Cramer’s V = 0.16).

## 4. Discussion

This study provides novel insights into patient perspectives on telemedicine adoption for cardiovascular risk management in Slovakia, addressing a significant research gap in Central and Eastern European healthcare contexts. The findings reveal several key patterns that differ from international trends and highlight specific challenges for telemedicine implementation in this region. The significance of this work lies not only in mapping the breadth of existing research, but also in clarifying how organizational culture has been underrepresented as a driver of LHS success. By systematically identifying cultural factors that facilitate or hinder learning processes, this article contributes a conceptual framework that can guide both researchers and practitioners. Specifically, it highlights areas where empirical evidence is sparse (e.g., cultural readiness assessments, leadership influence, and inter-professional trust) and offers practical implications for designing interventions that embed a learning-oriented culture into healthcare organizations. These claims go beyond descriptive mapping and provide actionable insights for future LHS development.

Our Slovak cohort demonstrated lower rates of daily blood pressure monitoring (21.2%) compared to international recommendations and studies from Western countries, where adherence rates of 40–60% have been reported [26,27,28,29]. This pattern contrasts sharply with findings from *Burke* et al., who achieved 78% adherence to daily self-monitoring in their weight loss mHealth trial, suggesting that engagement may be disease- and context-specific [30]. Similarly, while our study found moderate interest in telemedicine (69.8% overall, 27.4% strong interest), other mHealth monitoring studies like *Cossio* et al.’s leishmaniasis treatment monitoring achieved 85% patient participation rates, indicating that clinical urgency and disease severity may drive higher engagement [31].

The age-related disparities we observed (23.5% reluctance in patients >75 years vs. 7.1% in 18–30 years) align with broader mHealth literature, though they appear more pronounced than reported in other studies. *Chau* et al.’s validation of AI mHealth tools in older adults found better acceptance rates, but their intervention involved face-to-face support during implementation, suggesting that the delivery method significantly influences adoption in older populations [32]. Our findings indicate that email-based recruitment and self-directed technology use may exacerbate age-related barriers compared to supported implementation approaches.

The low awareness of LDL-C levels (73.8% unaware) in our cohort reflects a critical gap in cardiovascular risk management that exceeds rates reported in Western European studies, where typically 50–60% of patients are unaware of their cholesterol levels [33]. This finding underscores the potential value of integrated telemedicine platforms that could address multiple cardiovascular risk factors simultaneously.

The barriers identified in our study—cost (16.7%) and preference for traditional methods (19.8%)—align with global patterns reported in systematic reviews, though the relatively low percentage reporting barriers (66.3% reported no obstacles) suggests a potentially more favorable environment for telemedicine adoption in Slovakia compared to other Central European countries [17,18,19,20,21,22,23,24]. However, the clinical utility of these devices remains questionable given that most wearable devices lack validation for clinical blood pressure monitoring according to ESC guidelines [34,35].

In Slovakia, the Discovery study reported that 85.15% of monitored patients had hypertension, reflecting the high burden in clinical populations [34]. In a recent cohort, 67.4% of patients were diagnosed with hypertension, and 7.9% were managed with non-pharmacological therapy. These findings are consistent with regional data from neighboring countries, where hypertension prevalence among adults ranges from 30% to over 50%, depending on the population studied and the presence of comorbidities [35]. Current ESC guidelines for the management of elevated blood pressure and hypertension defines hypertension as systolic blood pressure ≥ 140 mm Hg or diastolic blood pressure ≥ 90 mm Hg, both in office, or use of antihypertensive medication [8]. Despite advances in detection and treatment, control rates remain suboptimal, especially in central and eastern Europe [1,34,35].

Despite these recommendations, adherence to proper HBPM technique and regular reporting remains suboptimal. Studies show that many patients do not measure their blood pressure correctly at home, lack adequate education on technique, or fail to share readings with their healthcare providers as recommended by guidelines. In a recent survey of rural US patients, only 51.5% checked their BP at home in the prior month, and just 29.2% shared readings with providers in the last six months. Inadequate patient education and lack of device access are common barriers [26,27].

In clinical cohorts, daily HBPM is uncommon; only a minority of patients measure daily, while a substantial proportion measure less than weekly or not at all. Factors associated with poorer adherence include younger age, smoking, and use of non-validated devices. Patients on pharmacological therapy and older adults are more likely to monitor BP regularly. These findings highlight the need for improved patient education, device access, and integration of HBPM data into clinical workflows to optimize hypertension management [26,27].

Telemonitoring and digital health interventions have demonstrated significant effectiveness in improving hypertension management, with robust evidence from recent randomized trials and meta-analyses. The HBPT-plus trial showed that pharmacist-led home BP telemonitoring resulted in a mean systolic BP reduction of 8 mmHg and diastolic reduction of 4 mmHg over usual care [14]. The *HOME BP* trial found a mean systolic BP reduction of 3.4 mmHg at one year with a digital intervention combining self-monitoring, data sharing, and guided management [15]. The E-health study and recent meta-analyses by *Kelly* et al. and *Sakima* et al. confirm these findings. It was reported that up to 90% of patients reached target BP with telemonitoring and active treatment adjustment, compared to 63–69% with standard care or education-only approaches [34]. Key factors for success include frequent home measurements, reliable data transmission to healthcare teams, and prompt, protocol-driven medication adjustments by professionals [35]. These components are often lacking in traditional care, as reflected in previous studies and local data showing poor BP follow-up and low rates of daily monitoring. In summary, digital interventions—when integrated with structured measurement, data sharing, and active clinical management—substantially improve blood pressure control and patient outcomes in hypertension.

Successful implementation of telemedicine for hypertension management depends on patients having access to clinically validated, upper-arm cuff-based blood pressure monitors, as recommended by the European Society of Hypertension, International Society of Hypertension, and other major societies. These guidelines emphasize that only validated, oscillometric, upper-arm devices should be used for home and telemonitoring, as wrist and finger devices, as well as most current wearable devices (including smartwatches, fitness bands, and rings), lack sufficient validation and accuracy for clinical decision-making [34,35].

Globally, access to home BP monitors varies widely: in high-income countries, 50–80% of hypertensive patients report having a home BP device, but rates are lower in low- and middle-income settings [17]. Digital and manual devices are most common; smart devices with wireless connectivity are increasingly available but remain less prevalent. Wearable BP monitors are under rapid development, but their clinical use is limited by insufficient validation, calibration drift, and lack of regulatory approval for most models [18]. Patient willingness to measure BP is a critical determinant of telemedicine success. Studies show that only a minority of patients measure BP daily, with adherence influenced by age, comorbidities, and whether they are on pharmacotherapy [19]. Telemonitoring programs that include patient education, frequent reminders, and integration with electronic health records (EHRs) significantly improve both measurement frequency and BP control [9,11].

Global trends indicate rising adoption of telemedicine and digital health tools, accelerated by the COVID-19 pandemic. Patient interest in telemedicine is high, but barriers include device cost, digital literacy, and interoperability with EHRs [20]. Integration of BP data into EHRs and use of digital platforms for clinician-patient communication are key to optimizing hypertension management and improving outcomes [9].

Widespread adoption of telemonitoring for hypertension management faces several persistent challenges. Organizational barriers include lack of standardized protocols, limited integration with electronic health records, and insufficient resources for program implementation and maintenance [17]. Many practices report inadequate IT infrastructure, human resources, and financial support, which can exacerbate disparities in engagement and care delivery [19]. Integration of telemonitoring platforms into routine clinical workflows is essential for success, but remains inconsistent across healthcare systems [20].

Economic barriers are significant, with high initial costs for devices, platforms, and training, as well as ongoing uncertainty regarding reimbursement models. The American Heart Association notes that reimbursement for telehealth services varies widely by payer and state, and payment parity with in-person visits is not universally supported, creating disincentives for clinicians and health systems. Cost-effectiveness and sustainability of telemonitoring programs require further evaluation in real-world settings [21].

Technical barriers include variable internet connectivity, unequal access to digital devices, and digital literacy gaps, particularly among older adults and socioeconomically disadvantaged populations [22]. These factors limit patient participation and the effectiveness of telemonitoring interventions. Proprietary platforms are used in some studies, but many clinicians and patients rely on general tools such as phone calls, email, or social messengers, which may delay clinical decision-making and reduce data quality [23].

Clinician perspectives reveal that while most recognize the value of digital tools for improving adherence and patient engagement, only a minority regularly use specialized telemonitoring platforms; most rely on general communication methods. Patient engagement is enhanced when telemonitoring is integrated with education, personalized support, and team-based care [24]. In some regions, such as Slovakia, a higher proportion of patients actively use specialized platforms and smart BP devices, suggesting that local context and targeted implementation strategies can improve uptake.

To address these barriers, evidence-based recommendations include: integrating telemonitoring into existing clinical infrastructure, providing devices on prescription, additional funding and training, simplifying IT tools, and adopting person-centered care approaches. Ongoing evaluation and adaptation of implementation strategies are necessary to ensure equitable access and sustained effectiveness of telemonitoring in hypertension management [19].

The proliferation of affordable, validated home blood pressure monitors and high rates of smartphone ownership have created an environment where mobile platforms for home blood pressure monitoring are increasingly accessible and widely used. The integration of information technology into clinical practice further supports the feasibility of remote hypertension management [9,36,37,38,39,40].

Despite the availability of numerous blood pressure tracking applications on major app stores, only a minority are suitable for clinical use. Most lack standardized protocols, evidence-based measurement guidance, and features such as reminders for morning/evening checks, automated calculation of average blood pressure over recommended monitoring periods, and direct physician involvement in design or feedback. The absence of clear standards for development and evaluation of mobile health applications is a recognized gap, as highlighted by the International Society of Hypertension and other major societies [41,42].

Recent randomized trials and meta-analyses indicate that enhanced self-measurement of blood pressure (SMBP) paired with connected smartphone applications can improve adherence and facilitate data sharing, but the impact on blood pressure control is modest unless combined with structured feedback, counseling, or clinician-driven interventions. Features most desired by patients include a simple user interface, personalized reminders, and direct feedback from clinicians, ideally without requiring complex data entry or navigation [43,44].

Emerging digital therapeutics and telemonitoring platforms show promise for improving hypertension management, especially when integrated with multidisciplinary care teams and robust analytics. However, device validation, interoperability, and long-term sustainability remain critical challenges [34]. The medical literature supports the continued development of evidence-based, user-friendly, and clinically integrated mobile platforms for home blood pressure monitoring [45].

More than half of Slovak patients present with both dyslipidemia and hypertension, necessitating comprehensive management of blood pressure and other cardiovascular risk factors [46,47]. In a recent cohort, 73.8% of respondents were unaware of their low-density lipoprotein cholesterol (LDL-C) level, and among those who knew their value, 45.7% had elevated LDL-C; several patients had levels between 5 and 8.2 mmol/L, indicating severe hypercholesterolemia. These findings are consistent with regional data, where only 24% of patients in Central and Eastern Europe achieve LDL-C targets recommended by the actual ESC guidelines, and three-quarters remain above goal despite lipid-lowering therapy [48].

Dyslipidemia remains a key modifiable risk factor for atherosclerotic cardiovascular disease and must be systematically addressed in risk management strategies [49]. Telehealth and digital interventions have shown positive to neutral impacts on lipid management, with improved patient satisfaction and easier implementation of multidisciplinary care, but barriers such as technology access, cost, and patient adherence persist [46,47,48,49,50,51,52]. Remote, algorithm-driven programs have demonstrated significant LDL-C reductions in high-risk populations, supporting their use as scalable strategies to close gaps in guideline-directed care.

Awareness and control of dyslipidemia can be improved through internet-based health management platforms, which have been shown to increase patient knowledge and control rates over time. Mobile phone-based interventions and telehealth dietary programs also show promise for improving medication adherence and modestly lowering LDL-C in adults at risk for cardiovascular disease [53,54]. However, persistent gaps in patient education and engagement highlight the need for ongoing innovation and integration of telemedicine into routine cardiovascular risk management.

## 5. Limitations

This study has several important limitations that significantly impact the generalizability of findings. The most critical limitation is the substantial selection bias introduced by the sampling methodology. Recruitment through email from a single cardiology clinic registry resulted in a highly selected population that differs markedly from the general Slovak population in several key ways.

First, clinic-based patients inherently represent individuals with established cardiovascular conditions who are already engaged with healthcare systems, making them unrepresentative of the broader population. Second, the email-based recruitment method strongly favored digitally literate respondents, as evidenced by the relatively high rates of smart device usage (28.6%) compared to national digital literacy statistics. The 9.9% response rate further compounds this bias, as respondents likely represent the most motivated and technology-engaged subset of the clinic population.

These biases likely resulted in significant overestimation of telemedicine interest and technology acceptance rates. The finding that 69.8% expressed interest in telemedicine may not accurately reflect Slovak population attitudes, particularly among older adults, rural populations, or those with lower digital literacy. Similarly, the relatively low percentage of reported barriers (66.3% reporting no obstacles) may reflect the pre-selected nature of the sample rather than actual population-wide attitudes.

Additional limitations include reliance on self-reported data, which may be subject to recall bias and social desirability bias, particularly regarding technology use and health behaviors. The cross-sectional design prevents assessment of causality or changes in attitudes over time. The questionnaire, while piloted, was not validated in larger populations, and some questions may not have captured the full complexity of patient attitudes toward telemedicine adoption.

Future research should employ population-based sampling methods, include face-to-face or telephone interviews to reduce digital literacy bias, and conduct longitudinal studies to assess actual technology adoption rates rather than stated intentions.

## 6. Conclusions

Slovakia demonstrates a potentially favorable environment for telemedicine implementation in cardiovascular risk management, as a significant portion of patients already utilize smart devices, wearables, and health monitoring applications. Nevertheless, substantial challenges remain, including limited overall uptake of daily monitoring practices, moderate rather than strong interest in telemedicine approaches, and age-related barriers to technology adoption. These barriers are influenced by patient age and diagnostic categories related to blood pressure.

Critically, willingness to participate in research should not be directly interpreted as readiness to adopt new technologies in everyday clinical practice, as demonstrated by the discrepancy between stated interest (69.8%) and strong interest (27.4%) in our study. Current evidence indicates that successful telemedicine adoption requires targeted patient education, tailoring of interventions to demographic and clinical characteristics, and systematic integration of patient feedback while strictly adhering to evidence-based practices and guideline recommendations.

The European Society of Cardiology and European Society of Hypertension emphasize the use of validated, cuff-based devices and standardized protocols for home blood pressure monitoring and telemonitoring platforms. Telemedicine interventions that integrate education, regular feedback, and multidisciplinary support have shown improvements in patient engagement, adherence, and clinical outcomes in hypertension and dyslipidemia management.

Optimizing telemedicine platforms in Slovakia should prioritize enhancing user-friendliness, ensuring access to validated devices, and embedding guideline-based workflows. Addressing patient-specific barriers and preferences, while maintaining rigorous clinical standards, will be essential for improving cardiovascular risk factor control and enhancing patient care outcomes.

## Data Availability

The original contributions presented in this study are included in the article. Further inquiries can be directed to the corresponding author.
